# Relationship between working conditions and mental health of migrants and refugees/asylum seekers vs. natives in Europe: a systematic review

**DOI:** 10.1007/s00420-023-01981-w

**Published:** 2023-07-13

**Authors:** Regina Herold, Marietta Lieb, Andrea Borho, Amanda Voss, Susanne Unverzagt, Eva Morawa, Yesim Erim

**Affiliations:** 1 Department of Psychosomatic Medicine and Psychotherapy, University Hospital of Erlangen, Friedrich-Alexander University Erlangen-Nürnberg (FAU), Schwabachanlage 6, 91054 Erlangen, Germany; 2 Institute and Outpatient Clinic of Occupational, Social, and Environmental Medicine, Friedrich-Alexander University Erlangen-Nürnberg (FAU), Erlangen, Germany; 3 Center of Health Sciences, Institute of General Practice and Family Medicine, Martin-Luther University of Halle-Wittenberg, Halle, Germany

**Keywords:** Migrants, Refugees, Asylum seekers, Mental health, Well-being, Europe

## Abstract

**Objective:**

Migrants and refugees/asylum seekers make up a significant proportion of the European workforce. They often suffer from poor working conditions, which might impact mental health. The main objective of this systematic review was to summarize and analyze existing research on working conditions of migrants and refugees/asylum seekers in European host countries and compare them to those of natives. Furthermore, the relationship between working conditions and mental health of migrants/refugees/asylum seekers and natives will be compared.

**Methods:**

Three electronic databases (PubMed/MEDLINE, PsycInfo and CINAHL) were systematically searched for eligible articles using quantitative study designs written in English, German, French, Italian, Polish, Spanish or Turkish and published from January 1, 2016 to October 27, 2022. Primary health outcomes were diagnosed psychiatric and psychological disorders, suicide (attempts), psychiatric and psychological symptoms, and perceived distress. Secondary health outcomes were more general concepts of mental health such as well-being, life satisfaction and quality of life. Screening, data extraction and the methodological quality assessment of primary studies by using the Newcastle–Ottawa Scale were done independently by two reviewers. The results of the primary studies were summarized descriptively. Migrants and refugees/asylum seekers were compared with natives in terms of the association between working conditions and mental health.

**Results:**

Migrants and refugees often face disadvantages at work concerning organizational (low-skilled work, overqualification, fixed-term contracts, shift work, lower reward levels) and social conditions (discrimination experiences) in contrast to natives. Most unfavorable working conditions are associated with worse mental health for migrants as well as for natives.

**Conclusions:**

Even if the results are to be taken with caution, it is necessary to control and improve the working conditions of migrants and refugees/asylum seekers and adapt them to those of the native population to maintain their mental health and thus their labor force.

**Supplementary Information:**

The online version contains supplementary material available at 10.1007/s00420-023-01981-w.

## Introduction

By mid-2020, nearly 281 million persons migrated internationally (McAuliffe and Triandafyllidou 2021). Among those, 30.5 million represented refugees and asylum seekers (United Nations High Commissioner for Refugees 2022). An “international migrant” is defined as a person who has changed his or her country of residence (United Nations 1998). Migration can be voluntary (because of work, family reunification or higher education) or forced (because of martial conflicts, persecution or catastrophes). Forced migrated persons are called refugees. Among them, those who have not yet been granted official refugee status are designated as asylum seekers (McAuliffe et al. 2019).

As high-income countries are the main migration destinations (McAuliffe and Khadria 2019), Europe is among the regions with the highest migrant rate (87 million) (United Nations 2020, 2021). Almost 12% of the European population can be identified as migrants (Kitimbo et al. 2022). Migrant workers account for 18.4% of the employed population in Northern, Southern and Western Europe and represent a substantial proportion of the labor force (International Labour Organization 2021).

Disadvantages in working conditions of migrants in contrast to natives have already been identified in the host countries. These include, e.g., migrants being mainly employed in low-skilled jobs (Arici et al. 2019; Kosyakova and Kogan 2022), receiving lower payment (Kosyakova and Kogan 2022; Moyce and Schenker 2018), facing greater risk of health hazards at work (Malhotra et al. 2013; Moyce and Schenker 2018; Yanar et al. 2018) and being more likely to suffer from exploitation and abuse (Moyce and Schenker 2018). Migrants from low- and middle-income countries (Hargreaves et al. 2019) as well as women (Kosyakova and Kogan 2022; Moyce and Schenker 2018; Rubiales-Gutiérrez et al. 2010) are particularly affected. These adverse working conditions might impact mental health (Hargreaves et al. 2019; Malhotra et al. 2013). In addition, migrants and especially refugees (due to the flight experience itself (Heeren et al. 2014; Kosyakova and Kogan 2022) and a subsequent asylum process in the host country that may be experienced as traumatic (Laban et al. 2004)) are generally considered particularly vulnerable in terms of their mental health. In various studies, migrants reported higher prevalence rates of post-traumatic stress disorder (PTSD) (Close et al. 2016) and lower general and mental health (Arici et al. 2019) compared to natives. Also among refugees and asylum seekers, a similar pattern emerged. They reported higher incidence of mental disorders such as PTSD and depression than natives (Blackmore et al. 2020; Close et al. 2016; Giacco and Priebe 2018).

While the European Union (EU) member states have taken a similar direction regarding migration and integration policies (Göbel 2019), they significantly differ from other Western countries such as the so-called classic immigration countries USA, Canada and Australia (Hoesch 2018). To the best of our knowledge, no systematic review has yet been conducted in this context with a particular focus on solely Europe. For this reason, there is a need to examine the working conditions of migrants and refugees/asylum seekers and their relationship with mental health in comparison to natives specifically for Europe. This is the only way to draw valid and realistic conclusions about working conditions of this population group in European countries. In addition, in order to obtain an up-to-date picture of the occupational circumstances of migrants and refugees/asylum seekers in Europe and their mental health, the focus should be on the period following the great wave of migration to Europe from 2014 (but especially in 2015 and 2016) (Grote 2018). This will allow mapping the direct impact of the refugee movement on the labor market as well as the current situation faced by migrants and refugees/asylum seekers in the European labor market.

## Objectives

The main objective of this systematic review is to descriptively elicit the relationship between working conditions and mental health of migrants and refugees/asylum seekers in European host countries. To better understand this relationship, working conditions of migrants and refugees/asylum seekers in European host countries are described and their relationship with mental health of migrants and refugees/asylum seekers is compared with those of natives.

## Materials and methods

This systematic review bases on a published protocol (CRD42021244840) (Herold et al. 2022). It follows the recommendations of the “Preferred Reporting Items for Systematic Reviews and Meta-Analysis” (PRISMA) (Page et al. 2021).

### Inclusion and exclusion criteria

The eligibility criteria of the included studies are described based on the Population, Exposure, Comparator and Outcome (PECO) framework.

#### Types of population (P)

Studies of internationally migrated first-generation migrants and refugees/asylum seekers of working age (15–70 years) (International Labour Organization 2021) were included. Participants must have been currently working (formally or informally) and residing in a European country (in the case of a longitudinal study, at least at the first measurement point). “European countries” were defined as all countries that were assigned to the continent of “Europe” from a geographical point of view (United Nations 2022). If crucial information about the inclusion criteria was missing, the corresponding authors were contacted. Articles for which clear age ranges were not known, even after contacting the study authors, were nevertheless included as the inclusion criterion of a currently working population should ensure that subjects were in the correct age range in most cases.

#### Types of exposure (E)

Original studies of the association of any working conditions with the mental health of migrants and refugees/asylum seekers in Europe were selected. Working conditions contained “organizational conditions” (e.g., income level, formality of work, work contract), “social conditions at work” (e.g., discrimination, prejudice, violence) or special issues such as “post-migration stressors migrants and refugees/asylum seekers in Europe are confronted with at the workplace” (Carlsson and Sonne 2018) (e.g., language barriers, mentality differences). The occurrence of these three topics in the primary studies was expected based on the existing literature and therefore introduced.

#### Types of comparators (C)

A comparison/control group was not obligatory.

#### Types of outcomes (O)

Primary outcomes were diagnosed psychiatric and psychological disorders, e.g., measured by common diagnostic procedures such as interviews (including suicide and suicide attempts), psychiatric and psychological complaints (e.g., anxiety, depression, somatoform disorders) and general distress. Secondary outcomes contained more general related constructs of mental health (e.g., well-being, quality of life, life satisfaction). Initially a more differentiated subdivision of the outcomes was planned (Herold et al. 2022). However, this was not done as this differentiation does not provide any added value for understanding the results on health outcomes.

Validated measurement instruments must have been used (at least in the original language).

### Study design

Quantitative studies such as randomized controlled trials, cohort studies, case–control studies and cross-sectional studies with and without control groups were included.

### Setting, language and publication status

No restrictions were defined with regard to the setting type. Studies written in English, French, German, Italian, Polish, Spanish and Turkish were considered. Only articles published in peer-reviewed journals were included. Unpublished studies as well as editorials, letters, “gray literature” such as conference abstracts, dissertations and non-peer review articles were excluded. Only full-text articles (independently evaluated by two reviewers (RH and FW/ML) using the “Strengthening the Reporting of Observational Studies in Epidemiology (STROBE-)Statement” (von Elm et al. 2007) were included.

### Information sources, time frame and search

PubMed/MEDLINE, PsycInfo and CINAHL were systematically and independently searched. In addition, reference lists of included studies and relevant reviews were screened. Furthermore, an unsystematic search on Google Scholar (www.scholar.google.de) was performed to find additional relevant studies. The first literature search for studies published on or after January 1, 2016 was independently conducted by two reviewers (RH and FW) on March 16, 2021. An update was performed on October 27, 2022 by two authors (RH and AB) to find all relevant studies published between January 1, 2016 onwards. The search strategy included the following three search term clusters: 1) terms related to the study population such as “migrant*” or “refugee*,” 2) terms related to working conditions such as “employ*” or “work*,” and 3) terms related to mental health outcomes such as “mental disorder*” or “well-being” (Supplement 1).

### Data management, data collection, selection process and extraction

**Fig. 1:**
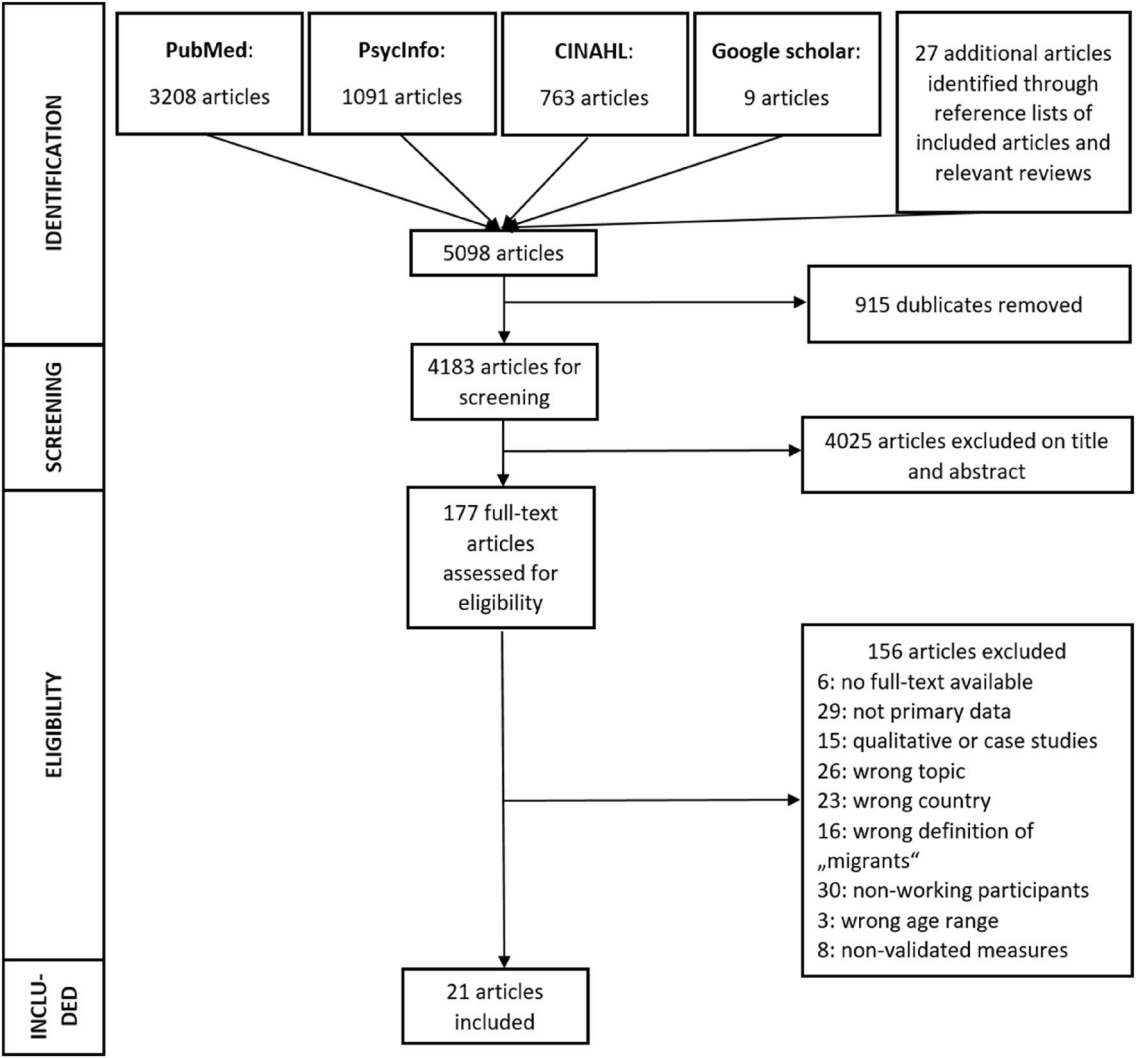
Flow diagram of study selection

EndNote X9 (The EndNote Team 2013) was used for study management. After duplicate detection and elimination, the two reviewers independently screened the titles and abstracts of the primary studies following the inclusion criteria. Relevant studies were then independently assessed for completeness. An additional unsystematic search was conducted and reference lists of relevant reviews and of included articles were searched for additional relevant studies. Disagreements were discussed between the two reviewers (RH and FW/ML). A third/fourth reviewer (YE and EM) was consulted if no agreement could be reached. A “Preferred Reporting Items for Systematic Reviews and Meta-Analyses “(PRISMA)” flowchart (Page et al. 2021) showing details of included and excluded studies at each study selection process stage is provided in Fig. 1.”

### Critical appraisal of the primary studies

Two reviewers independently appraised the quality of the included primary studies using the “Newcastle–Ottawa Quality Assessment Scale” (NOS) (Wells et al. 2000) adapted for cohort studies and cross-sectional studies. This rating contains a score range of 0–9 (0–3: “low quality,” 4–6: “moderate quality,” 7–9: “high quality” (Koshy et al. 2021)). Additionally, two reviewers independently rated the outcome measurement instruments according to whether they were used in the original language in which they were validated, or whether a translation or a culturally adapted version was used.

### Changes to the study protocol

In the conceptualization of this systematic review, it was planned to additionally investigate if migration status has an impact on the association between working conditions and mental health, comparing migrants with refugees/asylum seekers. Furthermore, it was planned to compare migrants and refugees/asylum seekers of different cultural backgrounds, using the concept of individualism and collectivism. Additionally, the working conditions and their influence on the mental health of migrants and refugees/asylum seekers in different host countries were to be compared. Since it seemed too extensive to address all these issues in one publication, we decided to split the topics and address only the issues mentioned under “objectives” in this article and publish the additional issues in another article.

## Results

### Study selection

The first literature search yielded 3722 articles in PubMed (*n* = 2349), PsycInfo (*n* = 802) and CINAHL (*n* = 571). The second literature search resulted in 1340 additional articles in PubMed (*n* = 859), PsycInfo (*n* = 289) and CINAHL (*n* = 192). In total, 915 duplicates were removed and 9 articles were found in the context of an unsystematic search so that 4183 items were identified for screening. A total of 177 full-text articles were read and 21 articles were included in the systematic review (Fig. 1).

### Study characteristics

An overview of the study characteristics of the included studies is provided in Table 1. The included studies and their research results are highlighted in more detail in Table 2.

**Table 1:** Study characteristics of the included primary studies

	Number of articles (*n* = 21)
Study design	
Cross-sectional	17
Cohort	4
Publication year	
2016	3
2017	2
2018	6
2019	3
2020	1
2021	4
2022	2
Study country	
Germany	5
Italy	4
Spain	3
Greece	2
Sweden	2
UK	2
Denmark	1
Finland	1
France	1
Participants	
Migrants	11
Migrants and natives	9
Migrants, refugees and natives	1
Country of origins of migrants and refugees	
Eastern Europe/Poland/Romania	5
Morocco	3
Ghana	2
Latin America/Colombia and Ecuador	2
China	1
Italy	1
Mixed and/or unknown	11
Occupations^a^	
Manufacturing industry (including construction)	4
Services	11
Agriculture, forestry, fishery	1
Mixed and/or unknown	8
Outcome types	
Primary	16
Secondary	1
Primary and secondary	4
Working conditions	
Work domain	5
Education–occupation match	4
Working position	2
Employment contract	9
Working schedule	9
Days off and holidays	1
Shift work	2
Physical demands	1
Work demands	4
Rewards	6
Work resources	5
Work strain/stress	4
Worksite size	1
Safety climate	1
Extreme working conditions	2
Leadership style	2
Discrimination	7

^a^Categorization based on the microcensus model (Destatis 2023)

**Table 2:** Characteristics and research results of the included primary studies

Authors, publication year (study country)	Study design (representativeness)	Total sample (size, medium age (SD^a^), percentage of women)	Migrant group(s) (size, percentage of the total sample, medium age (SD), percentage of women)	Country (/-ies) of origin (percentage of all migrants)	Control group(s) (size, kind of control group(s), percentage of the total sample, medium age (SD), percentage of women)	Occupational group(s) (percentage of the total sample)	Working conditions (measurement instrument(s))	Mental health outcome (measurement instrument(s))		Main results
								Primary	Secondary	
Braun et al. (2021) (Germany)	Cross-sectional study (not representative)	68 (10.3% women)	68 migrants (100%)	• Arab countries (57.4%) • Non-Arab countries (42.6%)	–	Urologists	• Contract type • Working schedule • Specialization • Work setting • Job position	Burnout (MBI^b^)	–	Having a permanent employment contract, working in managerial positions and working full-time as protective factors against the burnout dimension “Reduction of personal accomplishment”
Brendler-Lindqvist et al. (2022) (Sweden)	Cohort study (representative)	120,303 (50.2% women)	• 47,637 refugees (39.6%, 41.7% women) • 72,666 migrants (60.4%, 55.9% women)	• Eastern Europe, Russia and the post-Soviet republics (46.0%) • Western Europe, USA, Canada, Australia and New Zealand (11.5%) • Middle East (16.7%) • Horn of Africa and Sudan (3.9%) • South and Central America (4.5%) • East Asia (8.3%) • Other (9.0%)	–	–	Education–occupation match (SSYK96^c^)	Hospitalization for psychiatric diagnoses (ICD-10^d^)	–	Over- and underqualification as risk factors for hospitalization for psychiatric diagnoses
Capasso et al. (2016a) (Italy)	Cross-sectional study (not representative)	250 (*M* = 43.2 (SD = 4.3), 100% women)	250 migrants (100%)	Eastern Europe (100%)	–	Eldercare (100%)	• Job type • Contract type • Working schedule • Work characteristics (JCQ^e^) • Effort–Reward Imbalance (ERI^f^) • Work stress • Racial discrimination at work	• Interpersonal disorders (SCL-90-R^g^) • Anxious–depressive disorders (SCL-90-R^g^)	–	Negative association between high work demands and anxious–depressive as well as interpersonal disorders
Capasso et al. (2016b) (Italy)	Cross-sectional study (not representative)	900	700 migrants (77.8%)	• Eastern Europe (35.7%, *M* = 43.2, 100% women) • Morocco (35.7%, *M* = 40.8, 10% women) • Ghana (28.6%, *M* = 38.8, 9% women)	200 native Italians (22.2%)	• Eldercare (27.8%) • Factory workers (38.9%) • Masons (33.3%)	• Job type • Contract type • Monthly income • Working schedule • Work characteristics (JCQ^e^) • Effort–Reward Imbalance (ERI^f^)	• Interpersonal disorders (SCL-90-R^g^)• Anxious–depressive disorders (SCL-90-R^g^)	–	• Negative association between high rewards and anxious–depressive disorders for Moroccan and native factory workers • Positive association of high work demands and anxious–depressive disorders for native factory workers • Negative association of high rewards and anxious–depressive disorders for native masons • Negative association between high rewards and interpersonal disorders for Moroccan factory workers • Positive association between high demands and interpersonal disorders for native factory workers • Negative association between high work resources and interpersonal disorders for native factory workers • Positive association of high work demands and interpersonal disorders for Ghanaian and native masons • Negative association of high rewards and interpersonal disorders for native masons
Capasso et al. (2018a) (Italy)	Cross-sectional study (not representative)	900 (*M* = 41.4 (SD = 4.1), 32.6% women)	700 migrants (77.8%)	• Eastern Europe (35.7%) • Morocco (35.7%) • Ghana (28.6%)	200 native Italians (22.2%)	• Eldercare (27.8%) • Factory workers (38.9%) • Masons (33.3%)	• Job type • Contract type • Working schedule • Work characteristics (JCQ^e^) • Effort–Reward Imbalance (ERI^f^) • Work stress • Racial discrimination at work	• Interpersonal disorders (SCL-90-R^g^) • Anxious–depressive disorders (SCL-90-R^g^)	–	• Positive association between high work demands and interpersonal as well as anxious–depressive disorders for all workers • Negative association between high rewards and interpersonal disorders for all workers • Positive association between work stress and anxious–depressive disorders for all workers • Positive association of racial discrimination and interpersonal as well as anxious–depressive disorders for all workers • Higher risk for interpersonal disorders for Moroccan factory workers and Ghanaian masons • Higher risk for anxious–depressive disorders for native factory workers and Eastern European eldercare workers
Capasso et al. (2018b) (Italy)	Cross-sectional study (not representative)	250 (*M* = 40.8 (SD = 3.5), 10% women)	250 migrants (100%)	Morocco (100%)	–	Factory workers (100%)	• Job type • Contract type • Working schedule • Work characteristics (JCQ^e^) • Effort–Reward Imbalance (ERI^f^) • Work stress • Racial discrimination at work • Income level	• Interpersonal disorders (SCL-90-R^g^) • Anxious-depressive disorders (SCL-90-R^g^)	–	• Positive association between high work demands and work stress • Positive association between racial discrimination and interpersonal disorders • Negative association of high rewards and interpersonal as well as anxious–depressive disorders • Positive association between high work demands in addition to racial discrimination and interpersonal disorders • Higher risk for interpersonal disorders
Chatzea et al. (2018) (Greece)	Cross-sectional study (not representative)	176	85 migrants (48.3%)	International (100%)	91 native Greeks (51.7%)	Rescue workers (100%)	• Job type • Operation period • Shift duration • Number of dead adult refugees collected per rescue intervention • Number of dead children collected per rescue intervention	• Post-traumatic stress disorder (PCL-C^h^) • Burnout (MBI^b^)	Well-being (WBI-5^i^)	• Being a native Greek as predictor of PTSD, burnout and well-being • Operation period, duration of shifts, collection of dead adult bodies and collection of dead children bodies as significant predictors for PTSD, burnout and well-being
Espinoza-Castro et al. (2019) (Germany)	Cross-sectional study (not representative)	282 (39.4% women)	282 migrants (100%)	• Andean Community (55.7%) • Other South American Countries (26.2%) • Mexico and Central America (18.1%)	–	–	• Education–occupation match (ISCO-08^j^) • Violence at the workplace (physical violence and sexual harassment)	Common mental disorders (GHQ-12^k^)	–	• Overqualification as risk factor for common mental disorders • Positive association between violence at the workplace and common mental disorders
Espinoza-Castro et al. (2021) (Germany)	Cohort study (not representative)	189 (89.4% women)	189 migrants (100%, 89.4% women)	• Colombia (49.2%) • Mexico and Central America (27.0%) • South America without Colombia (12.7%) • Spain (9.5)	–	Au pairs (100%)	• Working schedule • Working on holidays • Days off per week • Existence of a contract • Extra hours • Additional jobs • Physical violence by host children • Verbal offenses • Violence at the workplace (physical violence and sexual harassment)	Depressive symptoms (PHQ-9^l^)	–	• Working more than 40 h per week as predictor for depressive symptoms • Suffering physical violence by host children as predictor for depressive symptoms
Gosselin et al. (2022) (France)	Cross-sectional study (representative)	19,211 (48.2% women)	898 migrants (4.7%)	• EU (31.7%) • Africa (43.3%) • Not EU, not Africa (24.9%)	18,313 native French (77.1%)	–	• Type of work domain • Contract type • Work sector • Worksite size • Night work • Job strain (JDCS model^m^) • Iso strain	Anxiety (GAD-Mini^n^)	–	• Positive association between job strain and anxiety disorder among native French and some migrant groups • Positive association between isostrain and anxiety among native French and some migrant groups
Holten et al. (2018) (Denmark)	Cohort study (not representative)	2947	111 migrants (3.8%, *M* = 46.0, 92.0% women)	• Europe (70.8%) • North America (0.9%) • South and Central America (3.8%) • Africa (7.5%) • Asia (11.3%) • Middle East (5.7%)	2836 native Danes (96.2%, *M* = 48.0, 98.0% women)	Eldercare (100%)	Transformational leadership (GTL^o^)	–	Well-being (WBI-5^i^)	Transformational leadership as a predictor for positive change in well-being for native Danes, but not for migrants
Hultin et al. (2016) (Sweden)	Cohort study (representative)	23,952 (56.1% women)	3349 migrants (14.0%)	• Nordic (36.2%) • Europe (27.3%) • Non-Europe (36.5%)	20,603 native Swedes (86.0%)	–	Education–occupation match (SSYK96^c^)	Common mental disorders (GHQ-12^k^)	–	Over- as well as underqualification no risk factor for psychological distress, neither for natives nor for migrants
Martynowska et al. (2020) (UK)	Cross-sectional study (representative)	551 (*M* = 33.0 (SD = 7.7), 75.0% women)	551 migrants (100%)	Poland (100%)	–	–	• Financial situation • Perceived change in attitude or behavior of supervisors • Perceived change in attitude or behavior of co-workers	Perceived stress (PSS-Mind Garden^p^)	• Psychological well-being (PWB^q^) • Life satisfaction (SWLS^r^)	• Positive association between negative change in attitude or behavior of supervisors or co-workers and perceived stress • Negative association between perceived stress and psychological well-being and life satisfaction
May et al. (2021) (Germany)	Cross-sectional (not representative)	81 (8.6% women)	81 migrants (100%)	–	–	Urologists	• Contract type • Working schedule • Specialization • Work setting • Job position	Burnout (MBI^b^)	–	Being employed as senior or chief physician as protective factor against the burnout dimension “Reduction of personal accomplishment “
Nie and Lämsä (2018) (Finland)	Cross-sectional study (not representative)	117 (41.0% women)	117 migrants (100%)	China (100%)	–	Employees in knowledge-based organizations (100%)	Paternalistic leadership of supervisor (PLS^s^)	Burnout (MBI^b^)	Leadership–Membership Exchange (LMX Scale)	• Negative association between benevolence (as an aspect of the paternalistic leadership style) and burnout • Positive association between benevolence and the social component of well-being (LMX) • Positive association between morality (as an aspect of the paternalistic leadership style) and the social component of well-being (LMX) • Positive association between authoritarianism (as an aspect of the paternalistic leadership style) and burnout
Ramos Villagrasa and García Izquierdo (2018) (Spain)	Cross-sectional study (not representative)	310	132 migrants (42.6%, *M* = 36.1 (SD = 10.0), 56.1% women)	• Latin America (72.7%) • Non-communitarian Europe (14.4%) • Africa (6.1%) • Other cultures from all over the world (6.8%)	178 native Spaniards (57.4%, *M* = 27.0 (SD = 9.4), 57.4%, 58.5% women)	• Service sector • Industry sector • Construction sector • Agriculture and fishing (0.8%)	• Type of work domain • Safety climate (Attitudes to Safety Scale)	Common mental disorders (GHQ-12^k^)	–	• Positive correlation between the communication and individual responsibility dimension of safety climate and well-being for migrants and natives • Positive correlation between the goal dimension of safety climate and well-being for migrants • Positive correlation between the individual responsibility dimension of safety climate and well-being for natives • Dimensions of safety climate no important predictor of well-being
Rhead et al. (2021) (UK)	Cross-sectional study (not representative)	931 (76% women)	328 migrants (35.27%)	–	603 natives (64.8%)	Healthcare workers (100%)	• Occupational group • Personal experience of discrimination at work • Witnessing discrimination at work • Personal experience of bullying/ harassment at work • Witnessing bullying/ harassment at work	• Depressive symptoms (PHQ-9^l^) • Generalized anxiety (GAD-7^t^) • Somatization symptoms (PHQ-15^u^)	–	• Positive association between personal experience of discrimination and bullying/harassment (but not witnessing) and probable anxiety or depression (even after adjusting for migration status) • Positive association between personal experience of bullying/harassment as well as witnessing discrimination and moderate or severe somatic symptoms (even after adjusting for migration status) • Positive association between personal experience of discrimination and somatic symptoms (but not after adjusting for migration status)
Ronda-Pérez et al. (2019) (Spain)	Cross-sectional study (not representative)	130	102 migrants (78.5%, 59.8% women)	Colombia, Ecuador	28 native Spaniards (21.5%, 50.0% women)	–	• Occupational social class • Working schedule • Formality of employment • Shif twork • Physical demands at the work place • Income level that precludes covering unforeseen expenses	Common mental disorders (GHQ-12^k^)	–	• Higher incidence of common mental disorders among natives than migrants independently of the working schedule (≤ 40 h per week or > 40 h per week) • Higher incidence of common mental disorders among natives than migrants with better working conditions (formal employment, no shift work, no physical demands, enough salary to cover unforeseen expenses)
Sifaki-Pistolla et al. (2017) (Greece)	Cross-sectional study (not representative)	176	85 migrants (48.3%, 20.0% women)	International (100%)	91 native Greeks (51.7%, 8.8% women)	Rescue workers (100%)	• Operation period • Duration of shifts • Number of dead refugees collected per rescue intervention • Number of dead children collected per rescue intervention	Post-traumatic stress disorder (PCL-C^h^)	–	• Positive association between operation period of more than 14 days as well as collection of more than one dead child per rescue intervention and higher probable PTSD • Positive association between daily shifts of more than four hours and the collection of more than six dead refugees per rescue intervention and higher probable PTSD among natives • Longer operation period, longer shift hours, collection of dead refugees and collection of dead children as major risk factors for probable PTSD
Virga and Iliescu (2017) (Spain)	Cross-sectional study (not representative)	477 (*M* = 32.0 (SD = 7.2), 29.0% women)	477 migrants (100%)	Romania (100%)	–	Blue-collar workers in construction work or agriculture (100%)	Job insecurity (JIS^v^)	• Burnout (MBI-General Survey^b^) • Mental health com-plaints (5-Item Scale)	–	Positive association between job insecurity and burnout as well as mental health complaints
Wassermann and Hoppe (2019) (Germany)	Cross-sectional study (not representative)	176 (*M* = 35.3 (SD = 7.9), 53.4% women)	176 migrants (100%)	Italy (100%)	–	–	• Working schedule • Perceived overqualification (SPOQ^w^)	Depressive symptoms (CES-D^x^)	Life satisfaction (SWLS^r^)	• Positive association between perceived overqualification and depressive symptoms • Negative association between perceived overqualification and life satisfaction

^a^Standard Deviation

^b^Maslach-Burnout Inventory

^c^Swedish Standard Classifications of Occupations (national adaptation to the International Standard Classification of Occupations (ISCO-88))

^d^International Classification of Diseases, version 10

^e^Job Content Questionnaire

^f^Effort–Reward Imbalance Scale

^g^Symptom Checklist 90 R

^h^Post-traumatic Stress Disorder Checklist-Civilian Version

^i^WHO-5-Well-being Index

^j^International Standard Classification of Occupations (ISCO-88)

^k^General Health Questionnaire-12

^l^Patient Health Questionnaire-9

^m^Karasek’s Job-Demand-Control-Support Model

^n^Generalized Anxiety Disorder, Mini International Neuropsychiatric Interview

^o^Global Transformational Leadership Scale

^p^Perceived Stress Scale-Mind Garden

^q^Scale of Psychological Well-being

^r^Satisfaction with Life Scale

^s^Paternalistic Leadership Scale

^t^Generalized Anxiety Disorder Scale-7

^u^Patient Health Questionnaire-15

^v^Qualitative job insecurity scale and quantitative Job Insecurity Scale

^w^Scale of Perceived Overqualification

^x^Short form of the Center for Epidemiological Studies Depression Scale

Two studies addressed different research questions in the same population (Capasso et al. 2016b, 2018a), while two other studies examined a subpopulation of the two mentioned before (Capasso et al. 2016a, 2018b). Two studies also made use of the same sample, considering different research aims (Chatzea et al. 2018; Sifaki-Pistolla et al. 2017). Two further studies used the same participant pool, which probably lead to some overlap (Braun et al. 2021; May et al. 2021).

### Quality appraisal

The results of the study quality appraisal of cross-sectional and cohort studies are presented in Tables 3 and 4. The quality of the studies showed scores with a minimum of 3 and a maximum of 8. Most of the cross-sectional studies were of moderate quality (*n* = 14), with some being of low quality (*n* = 3). The quality of one cohort study was high, another was considered low and the rest moderate (*n* = 2).

**Table 3:** Assessment of the methodological quality using the *“*Newcastle–Ottawa Quality Assessment Scale*”* for cross-sectional studies and evaluation of the validity of the questionnaire used

Authors, year	Selection			Comparability	Outcome			Total score (out of 9)	Validity of outcome measurement instruments
	Representativeness of the sample Maximum: *	Sample size Maximum: *	Comparability between respondents and non-respondents Maximum: *	Control of confounders Maximum: **	Assessment of the outcome Maximum: **	Statistical test Maximum: *	Ascertainment of the outcome measurement Maximum: *		
Braun et al. (2021				**		*	*	****^a^	Validated in German^b^
Capasso et al. (2016a)				**		*	*	****	Validated in Italian^b^
Capasso et al. (2016b)				**		*	*	****	Validated in Italian^b^
Capasso et al. (2018a)				**		*	*	****	Validated in Italian^b^
Capasso et al. (2018b)				**		*	*	****	Validated in Italian^c^
Chatzea et al. (2016)				**		*	*	****	All 3 validated in Greek and English^c^
Espinoza-Castro et al. (2019)				**		*	*	****	Validated in Spanish^c^
Gosselin et al. (2022)	*		*	**		*	*	******	Validated in French^c^
Martynowska et al. (2020)	*					*	*	***	All 3 validated in original language^d^
May et al. (2021)				**		*	*	****	Validated in German^c^
Nie and Lämsä (2018)				**			*	***	All 2 validated in English^c^
Ramos Villagrasa and García Izquierdo (2018)				**		*	*	****	Validated in Spanish^c^
Rhead et al. (2021)				**		*	*	****	All 3 validated in English^c^
Ronda-Pérez et al. (2019)				**		*	*	****	Validated in Spanish^c^
Sifaki-Pistolla et al. (2017)				**		*	*	****	Validated in Greek and English^c^
Virga and Iliescu (2017)				**			*	***	MBI validated in Romanian^b^, MHI-5 validated in English^d^
Wassermann and Hoppe (2019)				**		*	*	****	All 2 validated in Italian^d^

^a^Interpretation: 0–3 stars: low methodological quality, 4–6 stars: moderate methodological quality, 7–9: high methodological quality

^b^Not known if validated version was used

^c^Validated version was used

^d^At least validated in the original language

**Table 4:** Assessment of the methodological quality using the *“*Newcastle–Ottawa Quality Assessment Scale*”* for cohort studies and evaluation of the validity of the questionnaire used

Authors, year	Selection				Comparability	Outcome			Total score (out of 9)	Validity of outcome measurement instruments
	Representativeness of the exposed cohort Maximum: *	Selection of non-exposed cohort Maximum: *	Ascertain-ment of exposure Maximum: *	Presence of outcome of interest at start of study Maximum: *	Comparability of cohorts Maximum: **	Assessment of outcome Maximum: *	Follow-up time Maximum: *	Adequacy of follow-up Maximum: *		
Brendler-Lindqvist et al. (2022)	*	*	*		**	*	*	*	********^a^	International classification system
Espinoza-Castro et al. (2021)		*					*	*	***	Validated in Spanish^b^
Holten et al. (2018)		*	*		**		*	*	******	Validated in original language^b^
Hultin et al. (2016)	*	*			**		*	*	******	Validated in Swedish^c^

^a^Interpretation: 0–3 stars: low methodological quality, 4–6 stars: moderate methodological quality, 7–9: high methodological quality

^b^Validated version was used

^c^Not known if validated version was used

### Measurement tools

A variety of validated scales on mental health outcomes was used (Table 2). An evaluation of their validity can be found in Tables 3 and 4.

Concerning measurement tools for the assessment of working conditions, 13 studies used established questionnaires, partially validated. Organizational working conditions were explored (e.g., work domain, overqualification, employment contract) as well as social conditions (e.g., leadership style, discrimination). No post-migratory stressors migrants face at work were examined in primary studies.

### Sample characteristics

Eleven studies focused on one or more explicitly selected migrant group(s) from (a) particular country/-ies/regions of origin (Capasso et al. 2016a, b, 2018a, b; Espinoza-Castro et al. 2019, 2021; Martynowska et al. 2020; Nie and Lämsä 2018; Ronda-Pérez et al. 2019; Virga and Iliescu 2017; Wassermann and Hoppe 2019), while others examined migrants without a specific focus on their origin (Braun et al. 2021; Brendler-Lindqvist et al. 2022; Chatzea et al. 2018; Gosselin et al. 2022; Holten et al. 2018; Hultin et al. 2016; May et al. 2021; Ramos Villagrasa and García Izquierdo 2018; Rhead et al. 2021; Sifaki-Pistolla et al. 2017). One study took refugees into account (Brendler-Lindqvist et al. 2022). Seven studies examined migrants and refugees of different origins and reported the percentage distribution of their countries/regions of origin (Braun et al. 2021; Brendler-Lindqvist et al. 2022; Gosselin et al. 2022; Holten et al. 2018; Hultin et al. 2016; May et al. 2021; Ramos Villagrasa and García Izquierdo 2018). Further four studies did not report countries of origin at all (Chatzea et al. 2018; Nie and Lämsä 2018; Rhead et al. 2021; Sifaki-Pistolla et al. 2017). No study examined asylum seekers. Detailed sample characteristics can be found in Table 5.

**Table 5:** Sample characteristics

	Total	Migrants	Natives
Sample size ranges in primary studies, *n*	68–120,303	68–120,303	28–20,603
Sample size, *n* (%)	170,801 (100)	127,949 (74.91)	42,852 (25.09)
Gender, *n* (%)			
Men	80,606 (47.19)	61,125 (49.54)^a^	428 (12.84)^b^
Women	90,196 (52.81)	62,249 (50.46)^a^	2905 (87.16)^b^
Age range in years^c^	15–68	17–68	18–65
Mean age	41.40^d^	36.44^e^	46.41^f^
Migrants’ countries of origin^g^, *n* (%)			
Eastern Europe/Poland/Romania	1278 (53.14)		
Latin America	384 (15.97)		
Morocco	250 (10.40)		
Ghana	200 (8.32)		
Italy	176 (7.32)		
China	117 (4.86)		

Those 1576 participants who were examined several times were considered only once for the calculation of all characteristics

^a^For 4575 migrants, the gender distribution could not be calculated

^b^For 39,519 natives, the gender distribution could not be calculated

^c^The age range for 2947 participants was not reported

^d^The weighted total mean age could not be calculated for 165,264 participants

^e^The weighted mean age of migrants could not be calculated for 125,717 participants

^f^The weighted mean age of natives could not be calculated for 39,547 participants

^g^Eleven primary studies (*n* = 125,544 participants) did not focus on explicit regions/countries of origin

### Description of organizational conditions and their association with mental health

Different organizational conditions of migrants, refugees and natives were related to work domain, the match of education and occupation, working positions, employment contract, working schedule, days off and holidays, shift work, physical demands, work demands, rewards, work resources, work strain/stress, worksite size, safety climate and extreme working conditions.

#### Work domain

A total of five cross-sectional studies examined the work domain and reported a diverse picture. The majority of Latin American migrant workers of one study in Germany held a manual occupation (63%) (Espinoza-Castro et al. 2019). A study about Colombian and Ecuadorian migrants in Spain showed a higher frequency of employment in manual occupations for migrants than for natives (89% vs. 46%) (Ronda-Pérez et al. 2019). While among the examined healthcare workers in the UK, migrants were more likely than natives to be employed in jobs associated with lower socioeconomic status (healthcare assistants) (Rhead et al. 2021), in a Spanish study there were no differences in occupational sector among migrant and native workers (service sector: 73% vs. 69%, followed by construction: 15% vs. 21%, industry: 7% vs. 10% and agriculture/fishing: 1% vs. 0%) (Ramos Villagrasa and García Izquierdo 2018). In a representative study conducted in France, migrants worked mainly as skilled workers (29%: lower-level professionals, 20%: high-level professionals/managers, 17%: skilled clerical/sales/services, 16%: skilled laborers/factory workers) (Gosselin et al. 2022). Most of them worked in the private sector (74.5%) (Gosselin et al. 2022).

In terms of mental health, in one study from Spain independently of job domain natives had an increased risk for common mental disorders compared to Colombian and Ecuadorian migrants (non-manual: 53% vs. 9%, manual: 69% vs. 27%) (Ronda-Pérez et al. 2019). In a representative French study examining migrants the position of lower-level professional, skilled and unskilled clerical/sales/services, skilled and unskilled laborer/factory worker and farmer/entrepreneur showed a relationship with work strain and iso strain (when individuals are exposed to work strain but experience low social support) in contrast to the position of a high-level professional/manager. Further, working in the private sector was associated with work strain for migrants, but not with iso strain, compared to working in the public sector (Gosselin et al. 2022).

#### Education–occupation match

A total of two cross-sectional and two cohort studies examined education–occupation match. In a representative study examining a mixed sample of migrants and natives in Sweden, 8% of women and 6% of men worked below their skill level (overqualification) (total: 6%) (Hultin et al. 2016). In two representative Swedish studies and one non-representative German study, even a higher proportion of migrant workers was confronted with actual overqualification (12% (Brendler-Lindqvist et al. 2022), 22% (Hultin et al. 2016), 62% (Espinoza-Castro et al. 2019)), further 6% of migrant workers in Sweden were referred to as underqualified, while considerable 81% had a matching job with their education (Brendler-Lindqvist et al. 2022).

While in all of the cross-sectional studies education–occupation mismatch was associated with mental health problems in Germany ((actual overqualification associated with common mental disorders compared to having a job that matched one’s skill level for Latin American migrants (63% vs. 37%) (Espinoza-Castro et al. 2019), perceived overqualification associated with depressive symptoms and worse life satisfaction for Italian migrants (Wassermann and Hoppe 2019)), not all longitudinal studies could affirm this. In one longitudinal representative study from Sweden, actual over- and underqualification acted as risk factor of being hospitalized for mental or behavioral disorders for migrants (Brendler-Lindqvist et al. 2022). In another representative study, both actual over- and underqualification were not associated with psychological distress in a mixed sample of natives and migrants (Hultin et al. 2016).

#### Working position

A total of two cross-sectional studies from Germany studied working position. Among the examined migrant urologists, 26% worked in managerial positions (chief physicians, senior physicians) (Braun et al. 2021).

This occupational position was positively associated with a decrease in the burnout dimension “Reduction of personal accomplishment” in contrast to the position as specialist or assistant physician in two cross-sectional studies (Braun et al. 2021; May et al. 2021).

#### Employment contract

A total of eight cross-sectional studies and one cohort study examined employment contracts and showed a diverse picture between different European countries. A mixed sample of migrants and natives in Italy mainly had fixed-term contracts (48%) or temporary/casual jobs (42%) and only 9% had permanent contracts (Capasso et al. 2018a). In a representative study from France, a mixed sample mainly held permanent contracts or was employed as civil servants (86%), whereas 12% had fixed-term contracts or temporary work and 2% worked as apprentices (Gosselin et al. 2022). The examined Eastern European and Ghanaian migrants in Italy mainly held temporary/casual work or fixed-term contracts (97% (Capasso et al. 2016a, b), 98% (Capasso et al. 2016b)), the studied Moroccan migrants in Italy and migrants in Germany did so less often (44% (Braun et al. 2021), 54% (May et al. 2021), 66% (Capasso et al. 2016b, 2018b)). Among the examined native Italians, depending on the work domain, they had either fixed-term (83%) or permanent contracts (81%) (Capasso et al. 2016b). Regarding the frequency of (in)formality of jobs, 47% of the studied Spanish-speaking migrants in Germany reported not having signed an official contract (Espinoza-Castro et al. 2021). In a Spanish study among Colombian and Ecuadorian migrants, the frequency was lower, with no significant differences between migrants and natives (24% vs. 11%) (Ronda-Pérez et al. 2019).

In terms of mental health, in one study a permanent employment contract was associated with lower levels of the burnout dimension “Reduction of personal accomplishment” for migrant workers in a German cross-sectional study (Braun et al. 2021). However, in a representative French cross-sectional study, contract type did not show a relationship with work strain and iso strain for a mixed sample of migrants and natives (Gosselin et al. 2022). Among the examined Spanish-speaking migrants in Germany, those without official contract and those with contract suffered similarly from depressive disorders (25% vs. 28%). Also in the longitudinal course, no association between the existence of a contract and depressive symptoms was found (Espinoza-Castro et al. 2021). Colombian and Ecuadorian migrants and natives in informal employment did not differ in the risk of common mental disorders (42% vs. 61%) in a Spanish cross-sectional study. However, natives who were formally employed exhibited lower mental health than formally employed migrants (60% vs. 21%) (Ronda-Pérez et al. 2019).

#### Working schedule

A total of eight cross-sectional studies and one cohort study examined working schedule. The majority of the studied mixed samples in Italy and Spain worked full-time (64% of migrants and natives (Capasso et al. 2018a), 100% of migrants and natives (Ronda-Pérez et al. 2019)). Most examined migrants (72% of Italian migrants (Wassermann and Hoppe 2019), 93% of Eastern European migrants (Capasso et al. 2016a, b), 94% of migrants of different regions of origin (Braun et al. 2021), 95% of migrants of different regions of origin (May et al. 2021), 99% of Moroccan migrants (Capasso et al. 2016b, 2018b)) as well as all studied Italian natives (Capasso et al. 2016b) considered separately worked full-time. The examined Ghanaian migrants worked part-time (100% (Capasso et al. 2016b)). Among the studied Spanish-speaking migrants in Germany, almost one-fifth worked more than 40 hours per week (17%) (Espinoza-Castro et al. 2021). A Spanish study about Colombian and Ecuadorian migrants yielded even higher rates (68%) for those working more than 40 hours per week. Migrants did not differ from natives in this context (32%) (Ronda-Pérez et al. 2019). Most of the examined Spanish-speaking migrant workers (70%) in Germany worked extra hours (Espinoza-Castro et al. 2021).

In a German cross-sectional study, full-time work was related to lower scores on the burnout dimension “Reduction of personal accomplishment” compared to part-time work for migrant workers (Braun et al. 2021). Among the studied Spanish-speaking migrants in Germany, those who worked more than 40 hours per week suffered more frequently from depressive symptoms than those who did not (44% vs. 23%). In the longitudinal course working more than 40 hours per week also acted as risk factor for depressive symptoms for those migrants (Espinoza-Castro et al. 2021). Regardless of the number of weekly working hours, natives were more likely to suffer from common mental disorders than Colombian and Ecuadorian migrants in a Spanish cross-sectional study (≤ 40 h: 58% vs. 25%, > 40 h: 67% vs. 24%) (Ronda-Pérez et al. 2019). The examined Spanish-speaking migrants in Germany who worked extra hours did not differ from those who did not in terms of depressive symptoms (22% vs. 4%). Working extra hours did also not act as a predictor for depressive symptoms in the longitudinal course (Espinoza-Castro et al. 2021).

#### Days off and holidays

One cross-sectional study looked at days off and holidays. Almost one-third (28%) of the studied Spanish-speaking migrant workers in Germany reported one day off per week, the rest reported two days off. More than one-third (35%) of those migrants worked on holidays (Espinoza-Castro et al. 2021).

Those who reported one day off per week did not differ from those who had two days off in terms of depressive symptoms (33% vs. 24%). Those examined migrants working on holidays suffered more frequently from depressive symptoms than those who did not (43% vs. 19%). However, having only one day off or having to work on holidays did not act as predictors for depressive symptoms in the longitudinal course (Espinoza-Castro et al. 2021).

#### Shift work

Two cross-sectional studies analyzed shift work. The majority of a mixed sample of migrants and natives examined in a representative French study was not affected by night work (85%) (Gosselin et al. 2022). Colombian and Ecuadorian migrants examined in a study from Spain were more likely to work shifts than natives (40% vs. 14%) (Ronda-Pérez et al. 2019).

In a French representative study, night work was associated with work strain and iso strain for a mixed sample of migrants and natives (Gosselin et al. 2022). Natives and Colombian and Ecuadorian migrants who were examined in a Spanish study and who worked shifts did not differ in mental health (75% vs. 28%), while the studied natives without shift work were more affected by common mental disorders than migrants without shift work (58% vs. 23%) (Ronda-Pérez et al. 2019).

#### Physical demands

A cross-sectional study showed that the examined Colombian and Ecuadorian migrants and natives did not differ in the frequency of physical demands at work (46% vs. 36%) (Ronda-Pérez et al. 2019).

The studied Colombian and Ecuadorian migrants and native Spaniards with high physical demands did not differ significantly regarding mental health (30% vs. 60%), whereas natives without physical demands suffered from common mental disorders more often than migrants without physical demands (61% vs. 20%) (Ronda-Pérez et al. 2019).

#### Work demands

Four cross-sectional Italian studies investigated perceived work demands. Work demands include self-assessed overcommitment, effort and job demands (including time pressure, many interruptions, increased workload) (Capasso et al. 2018a). A mixed sample of migrants and natives with high work demands were more likely to suffer from interpersonal (insecurity in social contact, paranoid thoughts, compulsion, hostility) and anxious–depressive disorders (depression, somatization, anxiety) in an Italian study (Capasso et al. 2018a). Among all examined Eastern European, Moroccan and Ghanaian migrant workers with high work demands, 61% reported high levels of anxious–depressive as well as interpersonal disorders (Capasso et al. 2018a). Those studied Eastern European migrants considered separately with high work demands were in line with this and more likely to suffer from both disorder types (Capasso et al. 2016a, b), the examined Ghanaian migrants only from interpersonal disorders (Capasso et al. 2016b). The studied Moroccan migrants with high work demands did not suffer from either type of disorder (Capasso et al. 2016b, 2018b), but from increased perceived work stress (Capasso et al. 2018b). Only some of those Moroccan migrant workers who experienced racial discrimination at work, in addition to high work demands, suffered from interpersonal disorders (Capasso et al. 2018b). Some of the examined native Italian worker populations with high work demands were more likely to be affected by both types of disorders, others only by interpersonal disorders (Capasso et al. 2016b).

#### Rewards

Six cross-sectional studies examined rewards. Intrinsic/extrinsic rewards refer to self-assessed esteem reward and job security prospects reward (Capasso et al. 2016a, b, 2018a, b). For a mixed sample of natives and migrants of an Italian study, an association between high reward levels and a lower risk of interpersonal, but not of anxious–depressive disorders, was found. Among the group of all examined Eastern European, Moroccan and Ghanaian migrants, 65% of those with high reward levels reported low levels of interpersonal disorders (Capasso et al. 2018a). However, for the studied Eastern European and Ghanaian migrants high reward levels did not show any association with mental health (Capasso et al. 2016a, b), while an association was observed between high reward levels and better outcomes in both disorder types for the examined Moroccan migrants (Capasso et al. 2016b, 2018b). For all studied native groups, there was an association between high reward levels and lower risk for anxious-depressive disorders, but only for some groups there was also an association with interpersonal disorders (Capasso et al. 2016b).

Job insecurity as an unfulfilled reward could be identified as a risk factor for mental health as it showed a positive correlation with burnout symptoms and mental health complaints among Romanian migrant workers in a study from Spain (Virga and Iliescu 2017).

A special form of reward is an appropriate level of salary (Siegrist et al. 2004). The examined native Italian workers earned twice as much per month as their Moroccan and Ghanaian migrant counterparts (factory workers: 1200€ vs. 600€ (Capasso et al. 2016b, 2018b); masons: 800€ vs. 400€ (Capasso et al. 2016b)). The studied migrant Eastern European elderly care workers earned 800€ (Capasso et al. 2016b). Among the Polish migrants examined in a representative study from the UK, 81% rated their financial situation as good, while 17% reported a difficult situation (Martynowska et al. 2020). Colombian and Ecuadorian migrants were more likely than natives to report being unable to handle unforeseen expenses in a Spanish study (38% vs. 4%) (Ronda-Pérez et al. 2019). Being in a difficult financial situation was correlated with perceived stress in a representative study (Martynowska et al. 2020). While the examined natives and migrants who reported being unable to cope with unanticipated expenses did not differ significantly in terms of the risk of common mental disorders (0% vs. 31%), natives earning a sufficiently large amount of money were more likely to suffer from common mental disorders than the corresponding migrant groups (62% vs. 17%) (Ronda-Pérez et al. 2019).

#### Work resources

Five cross-sectional studies from Italy and France investigated work resources. Work resources contain social support at work and job control (Capasso et al. 2016a, b, 2018a, b). In France, a representative study showed that 15% of natives and 9 to 23% of migrants suffered from iso strain, with women being at risk (17% vs. 14% among men). Iso strain (as an indication of lacking social support) was associated with anxiety disorder among natives and some migrant groups (Gosselin et al. 2022).

The examined mixed sample of Eastern European, Moroccan and Ghanaian migrant and native workers in Italy with high work resources did not show lower risk of interpersonal or anxious–depressive disorders (Capasso et al. 2018a). For none of those migrant groups and almost none of those native groups did high work resources show an association with a lower risk for anxious–depressive and interpersonal disorders (Capasso et al. 2016a, 2016b, 2018b). Only one of those native groups with high work resources was less likely to suffer from interpersonal disorders (Capasso et al. 2016b).

#### Work strain/stress

Four cross-sectional studies examined work stress. In France, a representative study showed that 32% of natives and 20–44% of migrants suffered from work strain, with women being at risk (36% vs. 30% among men) (Gosselin et al. 2022).

Work strain was associated with anxiety disorder among natives and some migrant groups in a representative study from France (Gosselin et al. 2022). For the examined mixed sample of natives and Eastern European, Moroccan and Ghanaian migrants in Italy, perceived work stress was identified as a risk factor of anxious–depressive, but not interpersonal disorders (Capasso et al. 2018a). However, for all of those migrant groups work stress did not show any relationship with mental health (Capasso et al. 2016a, 2018b). Work stress did not act as a mediator in the relationship between work demands/work resources/rewards and interpersonal or anxious–depressive disorders for the studied Moroccan migrant workers in Italy (Capasso et al. 2018b).

#### Worksite size

One cross-sectional representative study examined worksite size. Almost half (48%) of migrant and native workers in France worked in a small workplace (≤ 50 persons), whereas more than one-third (35%) was employed in a workplace with 50–499 persons, 7% in a workplace with 500–999 persons and 11% in a workplace with ≥ 1000 workers.

Worksite size did not show an association with work strain or iso strain (Gosselin et al. 2022).

#### Safety climate

One cross-sectional study from Spain investigated safety climate. Safety climate includes three aspects: Communication (whether workers have been taught how to work safely), goals (whether minor accidents are considered normal) and individual responsibility (whether workers can influence safety on the job).

The studied migrants and natives did not differ in their perception of safety climate. Communication and individual responsibility showed a positive correlation with well-being in a mixed sample of migrants and natives. Considered separately, for migrants, but not for natives, a positive correlation between goals and well-being was evident. For natives, but not for migrants, a correlation between individual responsibility and well-being was found. However, in a multivariate design neither for migrants nor for natives, did the assessment of safety climate aspects show an association with well-being (Ramos Villagrasa and García Izquierdo 2018).

#### Extreme working conditions

Two cross-sectional studies from Greece focused on extreme working conditions of refugee rescuers such as the number of collected dead refugees or children per rescue intervention.

Traumatic working conditions were related to PTSD, burnout and worse perceived well-being for the examined native as well as migrant rescuers (Chatzea et al. 2018; Sifaki-Pistolla et al. 2017).

### Description of social conditions and their association with mental health

Different social conditions of migrants and natives were caused by leadership styles of their supervisors and discrimination in the work setting.

#### Leadership style

One cross-sectional and one cohort study examined leadership styles. A leadership style describes how a person influences others to follow the goal of organizations (Lawal 1993).

One kind of leadership style is referred to as the transformational leadership style. The transformational leadership style represents leaders who seek to satisfy the higher-order needs of their employees. Accordingly, it is characterized by leaders as well as employees motivating each other (Burns 1978). The examined migrants and natives rated their supervisors equally regarding transformational leadership style in a longitudinal study from Denmark. For both those natives and migrants, transformational leadership at baseline was associated with better well-being after two years. This association was not stated for migrant workers after well-being baseline control (Holten et al. 2018).

A cross-sectional study about Chinese migrants in Finland focused on the paternalistic leadership style. The dimensions that make up the paternalistic leadership style are called benevolence, morality and authoritarianism. Benevolence describes leaders’ behaviors that relate to an individual and comprehensive care for the work and welfare of subordinates, while morality is characterized by behavior that reflects the moral virtues of the supervisor. Authoritarianism describes the supervisor having authority over employees, demanding their full respect and deference (Cheng et al. 2004). The benevolence dimension of the paternalistic leadership style showed an association with Leadership–Membership Exchange (LMX, understood as well-being in terms of positive social relationships with supervisors) and a negative relationship with burnout symptoms for the studied migrants. Morality was also positively related to LMX. Authoritarianism was associated with higher burnout symptoms, but did not significantly relate to LMX (Nie and Lämsä 2018).

#### Discrimination

Five cross-sectional studies and one cohort study reported discrimination experiences. A representative study examining Polish migrants living in the UK showed that 14% noticed negative changes in attitudes toward them by colleagues, 5% positive changes and 81% no changes after BREXIT vote. Regarding changes in attitude on the part of supervisors, 9% reported negative changes, 7% positive changes and 84% no changes. Negative changes (from both colleagues and supervisors) were related to perceived stress (the extent to which life is uncontrollable, unpredictable and overloaded at the moment) which was associated with lower psychological well-being and life satisfaction in a cross-sectional study (Martynowska et al. 2020).

The cross-sectional studies about racial discrimination at work showed that among the examined Moroccan migrant workers in Italy, 26% reported having experienced racial discrimination (Capasso et al. 2018b). Racial discrimination was related to both interpersonal and anxious–depressive disorders in the studied mixed sample of Eastern European, Moroccan and Ghanaian migrant and native workers. Among the examined migrant workers, 52% of those with racial discrimination experiences suffered from high levels of anxious–depressive and 53% from high levels of anxious–depressive as well as interpersonal disorders (Capasso et al. 2018a). While racial discrimination showed an association with interpersonal disorders but not with anxious–depressive disorders for the studied Moroccan migrant groups (Capasso et al. 2018b), no association with mental health was shown for the examined Eastern Europeans (Capasso et al. 2016a). Some of those studied Moroccan migrants who perceived high work demands in addition to racial discrimination were more likely to suffer from interpersonal disorders (Capasso et al. 2018b).

In a cross-sectional study from the UK, among a mixed sample of migrants and natives, 21% reported personal discrimination experiences from a manager or colleagues in the last 12 months, while 44% reported personal bullying/harassment experiences by colleagues. Women were more likely to suffer discrimination (24% vs. 11%) and harassment (46% vs. 36%) than men. Migrants were both more likely to personally experience discrimination and bullying/harassment and to witness colleagues being victims of discrimination than natives. Personal experiences of discrimination and bullying/harassment, but not witnessing them, were associated with probable generalized anxiety or depression, even after controlling for sociodemographic data such as migration status. Personal discrimination and bullying/harassment experiences as well as witnessing discrimination and bullying/harassment, were associated with moderate or severe somatic symptoms, even after controlling for sociodemographic variables such as migration status (Rhead et al. 2021).

Among the examined Latin American and Colombian and Ecuadorian migrants in Germany, 14% (Espinoza-Castro et al. 2019) and 6% (Espinoza-Castro et al. 2021) reported physical violence or sexual harassment in the workplace. Furthermore, 30% and 23% of the studied migrant au-pairs experienced physical violence by host children and verbal offenses at the workplace, respectively. Migrants who were confronted with violence at work, physical violence by host children and verbal offenses suffered more frequently from depressive symptoms than those who did not (violence: 100% vs. 24%, physical violence: 52% vs. 17%, verbal offenses: 45% vs. 20%) (Espinoza-Castro et al. 2021). Consistent with these findings, a cross-sectional study showed that workplace violence was associated with common mental disorders (Espinoza-Castro et al. 2019), while in the longitudinal course workplace violence as well as verbal offenses did not act as risk factors for depressive symptoms. However, physical violence by host children was shown to be a risk factor for depressive symptoms over the longitudinal course (Espinoza-Castro et al. 2021).

## Discussion

This systematic review provides an overview of the working conditions of migrants and refugees in comparison with natives in European host countries and examines their relationship with mental health. Migrants and refugees are disadvantaged with regard to some of the working conditions addressed in the primary studies in contrast to natives. In terms of organizational conditions, migrants tend to work more often in jobs that are considered low-skilled or associated with lower socioeconomic status (based on the evaluation of one representative and four non-representative primary studies), more often face education–occupation mismatch (especially overqualification) (two representative and one non-representative primary studies), tend to hold fixed-term employment contracts more often (one representative and eight non-representative primary studies), are more likely to work in shifts (one representative and one non-representative primary study), and are disadvantaged in terms of rewards (financial compensation) (one representative and three non-representative primary studies). In terms of social conditions, migrants often face discrimination at work (negative attitudes toward them, racial discrimination, physical and verbal violence or sexual harassment), partly more often than natives (one representative and four non-representative primary studies). Migrants face similar conditions in terms of working schedule (mainly full-time) (nine non-representative primary studies), physical demands (one non-representative primary study), work resources (social support at work, job control) (one representative primary study), work strain/stress (one representative primary study), safety climate (one non-representative primary study) and leadership style of supervisors like natives (two non-representative primary studies).

Regarding mental health, a lower-skilled employment (based on the evaluation of one representative and one non-representative primary study), high work demands (four non-representative primary studies), night shift (one representative and one non-representative primary study) and discrimination (one representative and six non-representative primary studies) show a negative association with mental health for migrants and natives. Education–occupation mismatch was found to be negatively related to mental health as well, especially for migrants (two representative and two non-representative primary studies). Extreme working conditions faced by rescuers, involving severe human suffering and death, also reveal a negative association with mental health for both migrants and natives (two non-representative primary studies). Work stress is partly related to worse mental health for migrants (one representative and three non-representative primary studies). For migrants and natives, high rewards (one representative and six non-representative primary studies) and in parts work resources (four non-representative primary studies) are positively associated with mental health. Permanent employment contracts act as a protective factor for mental health for migrants (one representative and three non-representative primary studies). Full-time work is associated with better mental health among migrants as well, whereas an obligatory workweek of more than 40 hours acts as a risk factor (three non-representative primary studies). Different leadership styles are also related to mental health. A transformational leadership style has positive effects on mental health for natives, but not for migrants (one non-representative primary study). Partial aspects of a paternalistic leadership style demonstrate a positive association with mental health for migrants, some other aspects show negative relationships (one non-representative primary study).

The reason for most of these disadvantages of migrants and refugees in the labor market is due to the lack of recognition of vocational training from the country of origin as well as structural discrimination. Migrants and refugees are more likely to hold low-skilled jobs with a lower socioeconomic status, as they are heavily affected by overqualification. This is due to the fact that their training and degrees are frequently not recognized in the European host country (Brücker et al. 2021; Kosyakova and Kogan 2022). However, since especially in European countries such as Germany a recognized vocational qualification is mandatory for practicing a higher profession, migrant and refugee/asylum seeker workers are forced to take up menial jobs for which no vocational training is required (Brücker et al. 2021). An employment below the level of the own socioeconomic status is associated with lower levels of control and autonomy in the work setting (Smith and Frank 2005) which, in turn, is associated with increased distress (Chen et al. 2010; Smith and Frank 2005). The fact that migrants and refugees are more likely to hold fixed-term employment contracts, are more likely to work in shifts, and are disadvantaged in terms of rewards (financial compensation) is partly due to unrecognized educational degrees from abroad as well, which force migrants and refugees/asylum seekers to accept jobs with poor working conditions. To counteract this phenomenon, European host countries should look more closely which education acquired abroad is comparable to that in the host country and can be recognized. This could potentially allow many migrants and refugees/asylum seekers in European host countries to gain a foothold in the profession they already had in their country of origin and would lead to a better fit between profession and skills in the host country. Furthermore, these unfavorable working conditions show the openly practiced structural discrimination in host countries, which proves that migrants and refugees/asylum seekers do not have the same chance to get employments with similar working conditions.

In addition to political structural discrimination in labor policy, migrants and refugees were also affected by interactional discrimination, ranging from negative attitudes toward them to physical and sexual violence in the workplace. The frequent mention of discrimination experiences by migrants and refugees/asylum seekers in host countries, especially of an interactional nature, as well as its status as one of the main risk factors for the mental health of this population (Arici et al. 2019; Fernandes and Pereira 2016; Gray et al. 2021) highlight the widespread and thus high relevance of this phenomenon. To increase productivity of the work team as well as maintain mental health of migrant and refugee/asylum seeker workers, companies in host countries should provide targeted team building programs for their co-workers to improve social and emotional interaction among colleagues and supervisors in the multiethnic team, to provide fair treatment and equal opportunities and minimize prejudice as well as discriminatory judgements.

Since most primary studies did not examine gender differences, no conclusion can be drawn on this aspect. The few primary studies that did consider gender differences indicate that working women often suffer from worse working conditions (especially in terms of discrimination (Rhead et al. 2021), work strain and iso strain (Gosselin et al. 2022)) than men and are more mentally burdened (Chatzea et al. 2018; Sifaki-Pistolla et al. 2017). This is consistent with existing literature (Bildt and Michelsen 2002; Moyce and Schenker 2018; Rubiales-Gutiérrez et al. 2010). The particular vulnerability of women highlights the need for future research to also explore gender differences in terms of their working conditions and their association with mental health in relation to migration background.

Reviews on working conditions and their association with mental health of migrants and refugees/asylum seekers in some European and non-European countries are mostly in line with our results (Arici et al. 2019; Hargreaves et al. 2019; Sterud et al. 2019). Thus, Europe does not seem to differ much in terms of migration policies and integration of migrants and refugees/asylum seekers into the labor market. Whereas in the existing reviews, physical disadvantages in the form of more frequent occupational accidents or injuries came to the fore, this was rarely, if ever, addressed in the primary studies included in our review.

### Strengths and limitations

An important strength of the present systematic review is found in the very strict inclusion and exclusion criteria, especially with regard to the definition of “migrant.” Internationally, no common definition of “migrant” exists. In Germany, for example, the common definition is that “migrant” includes both first- and second-generation migrants (Destatis 2020), whereas in other countries “migrant” only includes first-generation migrants. This definition ultimately leads to the fact that second-generation migrants would be understood as natives. These different definitions would have resulted in unpossible comparisons between studies. For this reason, we decided to use a single definition of migrant as first-generation migrant. However, this resulted in the fact that studies without a clear definition of “migrant” and without the necessary information from the contacted author were excluded. During the literature search, we found relevant studies in which not all subjects were currently employed. The fact that we excluded those studies can be seen as an advantage, as this ensured that the included participants were able to make a valid and realistic statement about the circumstances at work. However, the exclusion of these studies resulted in a relatively large primary study loss (*n* = 30). Nevertheless, it is important to note that longitudinal studies were included in which subjects were working at the time of the first survey but then became unemployed during the follow-up. In this case, it was more important for us to include relatively rare and therefore valuable longitudinal studies as they allow causal inferences, than to adhere to our very strict inclusion criterion. Participants who were working at the first measurement point should still have insight into working life and therefore also be able to make reliable statements about their working conditions. Another strength can be seen in our linguistic expertise. We were able to include primary studies in seven European languages, which should have covered a wide range of relevant primary studies, especially since it can be assumed that most relevant studies from Europe are published mainly in these languages.

However, the present systematic review also has some limitations. One limitation is the type of articles included in the search. “Gray literature” was explicitly excluded. For this reason, a publication bias could not be prevented (Scherer et al. 2007). However, because “gray literature” often does not report in-depth relevant details that would have been necessary (O'Connor et al. 2014), it cannot be assumed that much relevant content was lost. Furthermore, it should not be ignored that most of the studies included in this review (*n* = 17) cannot be considered representative. For this reason, the results should be interpreted very cautiously. Accordingly, from this review it can be concluded that there is a large gap in representative data in this research area, which should be considered in future studies. It should also be noted that fewer studies have examined the relationship between working conditions and mental health among natives than among migrants. Because of this disparity, comparisons of the relationship between working conditions and mental health should be interpreted with caution. Furthermore, a look at the sample sizes of the primary studies shows relatively small total group sizes in some cases (*n* = 68 migrants (Braun et al. 2021), *n* = 81 (May et al. 2021)) as well as native and migrant groups that differ greatly in size (e.g., 2836 natives vs. 111 migrants (Holten et al. 2018), 28 natives vs. 102 migrants (Ronda-Pérez et al. 2019)). Therefore, due to the insufficient power in some primary studies, small or moderate effects probably could not have been detected. For this reason, these results in particular should be viewed with caution. A further limitation is the fact that the population we examined (migrants as well as refugees/asylum seekers) is a so-called hard-to-reach population. This is due to the lack of national registries and the difficulty of recruiting this population due to their lower willingness to participate in studies (Mesa-Vieira et al. 2022). As a result, despite an extensive systematic search, only one primary study could be found that investigated refugees in the work setting (a comparison between migrants and refugees/asylum seekers regarding their working conditions and their association with mental health is planned as part of a systematic review in a future publication). The fact that migrants are a difficult to recruit participant group for research ultimately leads to a fragmentary picture of persons in the labor market who have migrated to Europe (Mesa-Vieira et al. 2022). An additional limitation was revealed by the fact that some primary studies did not examine a specific migrant group, but heterogeneous migrant groups consisting of people from different countries. This means that no information exists on whether refugees were among them. This makes it difficult to determine whether the results refer to voluntary migrants only or whether they include a proportion of refugees. Another limitation is the methodological mostly moderate to low quality of the primary studies among the Newcastle–Ottawa Scale (Wells et al. 2000). The main weaknesses of the primary studies were a lack of representativeness of the sample, a lack of justification of the sample size, insufficient comparability of respondents and non-respondents, and the assessment of outcomes (since self-report measures were used). This shows that high-quality research in the field of migrants in the work setting is lacking and more high-quality research should be conducted in this field in the future. Furthermore, a glance at the outcome measurement instruments reveals a heterogeneous picture. Mental health outcomes such as depression symptoms or well-being were measured with different instruments, which makes comparability between the studies difficult. However, the explicit exclusion of measurement instruments that were not validated at least in the original language should ensure that only reliable and valid measurement instruments were used. This at least allows reliable statements and conclusions. However, the bias susceptibility of self-report measures should always be taken into account, as subjects may show specific response tendencies, for example, due to social desirability (Furnham and Henderson 1982). With regard to working conditions, there were no requirements for the validity of the measurement instruments. In part, different measurement instruments were also used here to query the same construct which makes comparability between the studies difficult. However, our aim was to describe working conditions as broadly as possible. For this reason, a high heterogeneity of the survey methods of working conditions is to be seen as an advantage.

## Conclusion

Overall, our systematic review showed a lack of studies on working conditions of migrants and refugees/asylum seekers in Europe and their impact on mental health. Migrants and refugees experience organizational disadvantages such as the tendency of more frequent employment in low-skilled jobs with lower socioeconomic status, overqualification, the tendency of more frequent employment under fixed-term contracts and in shift work, and lower reward levels as well as social disadvantages such as interactional discrimination compared to natives in all European host countries. For migrants and natives, most unfavorable working conditions were associated with worse mental health. Thus, labor law in European countries should pay attention to working conditions and control them to ensure equal opportunities for migrants and natives. Partially, an explicit specification of the laws for handling migrant workers would be necessary. As migrants make up a large part of the European workforce, maintaining their mental health by improving their working conditions should be a long-term goal. At political level this could be achieved, e.g., through broader governmental recognition of education from abroad. At company level this could be realized through anti-discrimination measures, team building programs as well as measures of workplace health promotion like providing education on work-related health hazards and information on employee rights in different languages.

## Supplementary Information

Below is the link to the electronic supplementary material.

Supplementary file1 (DOCX 14 KB)
