# Supplementary material for: Relationship between working conditions and mental health of migrants and refugees/asylum seekers vs. natives in Europe: a systematic review
Source: Int Arch Occup Environ Health. 2023 Jul 13;96(7):931–63. doi: 10.1007/s00420-023-01981-w (PMC10361874; doi:10.1007/s00420-023-01981-w)
Supplement: Supplementary file 1 — Supplementary file1 (DOCX 14 KB) [file 420_2023_1981_MOESM1_ESM.docx]

Relationship between working conditions and mental health of migrants and refugees/asylum seekers in Europe

Regina Herold, Marietta Lieb, Andrea Borho, Amanda Voss, Susanne Unverzagt, Eva Morawa and Yesim Erim

Regina Herold, Department of Psychosomatic Medicine and Psychotherapy, University Hospital of Erlangen, Friedrich-Alexander University Erlangen, Schwabachanlage 6, 91054 Erlangen; corresponding author, [regina.herold@uk-erlangen.de](mailto:regina.herold@uk-erlangen.de), ORCID-ID: 0000-0002-9551-6661

International Archives of Occupational and Environmental Health

**SUPPLEMENT 1** Exact search strategies of PubMed/MEDLINE, PsycINFO and CINAHL, 15/03/2021

**PubMed/MEDLINE**

(("transients and migrants"[MeSH Terms] OR "emigrants and immigrants"[MeSH Terms] OR "refugees"[MeSH Terms] OR "minority groups"[MeSH Terms] OR "human migration"[MeSH Terms] OR "migra*"[Title/Abstract] OR "emigra*"[Title/Abstract] OR "immigra*"[Title/Abstract] OR "refugee*"[Title/Abstract] OR "asylum seeker*"[Title/Abstract] OR "foreign*"[Title/Abstract] OR "resettl*"[Title/Abstract] OR "guest worker*"[Title/Abstract] OR "displaced person*"[Title/Abstract]) AND ("employment"[MeSH Terms] OR "work"[MeSH Terms] OR "occupations"[MeSH Terms] OR "employ*"[Title/Abstract] OR "work*"[Title/Abstract] OR "occupation*"[Title/Abstract] OR "vocation*"[Title/Abstract] OR "jobs"[Title/Abstract] OR "job"[Title/Abstract]) AND ("mental health"[MeSH Terms] OR "mental disorders"[MeSH Terms] OR "stress, psychological"[MeSH Terms] OR "mental fatigue"[MeSH Terms] OR "depressive disorder"[MeSH Terms] OR ("depressive disorder"[MeSH Terms] OR "depression"[MeSH Terms]) OR "quality of life"[MeSH Terms] OR "well-being"[Title/Abstract] OR "wellbeing"[Title/Abstract] OR "quality of life"[Title/Abstract] OR "life satisfaction"[Title/Abstract] OR "mental*"[Title/Abstract] OR "psychiatric*"[Title/Abstract] OR "psychological*"[Title/Abstract] OR "anxi*"[Title/Abstract] OR "depress*"[Title/Abstract] OR "burnout"[Title/Abstract] OR "burn-out"[Title/Abstract] OR "somatization"[Title/Abstract] OR "somatisation"[Title/Abstract] OR "somatoform"[Title/Abstract] OR "pain disorder"[Title/Abstract] OR "somatic symptom disorder"[Title/Abstract] OR "suicid*"[Title/Abstract] OR "insomnia"[Title/Abstract] OR "emotional stress"[Title/Abstract] OR "distress"[Title/Abstract])) NOT ("USA"[Title/Abstract] OR "United States"[Title/Abstract] OR "Canada"[Title/Abstract] OR "Australia"[Title/Abstract] OR "migraine"[Title/Abstract] OR "cancer"[Title/Abstract] OR "qualitative stud*"[Title/Abstract]) Filters: English, French, German, Italian, Polish, Spanish, Turkish, from 2016/1/1 - 2022/10/27

**PsycINFO via EBSCOhost:**

(TI emigra* OR TI immigra* OR TI refugee* OR TI "asylum seeker*" OR TI foreign* OR TI resettl* OR TI "guest worker*" OR TI "displaced person*“ OR AB migra* OR AB emigra* OR AB immigra* OR AB refugee* OR AB "asylum seeker*" OR AB foreign* OR AB resettl* OR AB "guest worker*" OR AB "displaced person*“) AND (TI employ* OR TI work* OR TI occupation* OR TI vocation* OR TI jobs OR TI job OR AB employ* OR AB work* OR AB occupation* OR AB vocation* OR AB jobs OR AB job) AND (TI "mental health" OR TI "psychosocial health" OR TI "mental disorder*" OR TI "psychosocial disorder*" OR TI distress OR TI "emotional stress" OR TI "well-being" OR TI wellbeing OR TI "quality of life" OR TI "life satisfaction“ OR AB "mental health" OR AB "psychosocial health" OR AB "mental disorder*" OR AB "psychosocial disorder*" OR AB distress OR AB "emotional stress" OR AB "well-being" OR AB wellbeing OR AB "quality of life" OR AB "life satisfaction") NOT (TI USA OR TI "United States" OR TI Canada OR TI Australia OR TI migraine OR TI cancer OR TI "qualitative stud*" OR AB USA OR AB "United States" OR AB Canada OR AB Australia OR AB migraine OR AB cancer OR AB "qualitative stud*") Limiters - Publication Year: 2016-2021 Expanders - Apply equivalent subjects Narrow by Language: - turkish Narrow by Language: - french Narrow by Language: - italian Narrow by Language: - spanish; castilian Narrow by Language: - german Narrow by Language: - english Search modes - Boolean/Phrase

**CINAHL via EBSCOhost:**

(TI migra* OR TI emigra* OR TI immigra* OR TI refugee* OR TI "asylum seeker*" OR TI foreign* OR TI resettl* OR TI "guest worker*" OR TI "displaced person*“ OR AB migra* OR AB emigra* OR AB immigra* OR AB refugee* OR AB "asylum seeker*" OR AB foreign* OR AB resettl* OR AB "guest worker*" OR AB "displaced person*“) AND (TI employ* OR TI work* OR TI occupation* OR TI vocation* OR TI jobs OR TI job OR AB employ* OR AB work* OR AB occupation* OR AB vocation* OR AB jobs OR AB job) AND (TI "mental health" OR TI "psychosocial health" OR TI "mental disorder*" OR TI "psychosocial disorder*" OR TI distress OR TI "emotional stress" OR TI "well-being" OR TI wellbeing OR TI "quality of life" OR TI "life satisfaction" OR AB "mental health" OR AB "psychosocial health" OR AB "mental disorder*" OR AB "psychosocial disorder*" OR AB distress OR AB "emotional stress" OR AB "well-being" OR AB wellbeing OR AB "quality of life" OR AB "life satisfaction“) NOT (TI USA OR TI "United States" OR TI Canada OR TI Australia OR TI migraine OR TI cancer OR TI "qualitative stud*" OR AB USA OR AB "United States" OR AB Canada OR AB Australia OR AB migraine OR AB cancer OR AB "qualitative stud*") Limiters - Published Date: 20160101-20211231 Expanders - Apply equivalent subjects Narrow by Language: - spanish Narrow by Language: - german Narrow by Language: - english Search modes - Boolean/Phrase
